# Real-time data processing for serial crystallography experiments

**DOI:** 10.1107/S2052252524011837

**Published:** 2025-01-01

**Authors:** Thomas White, Tim Schoof, Sergey Yakubov, Aleksandra Tolstikova, Philipp Middendorf, Mikhail Karnevskiy, Valerio Mariani, Alessandra Henkel, Bjarne Klopprogge, Juergen Hannappel, Dominik Oberthuer, Ivan De Gennaro Aquino, Dmitry Egorov, Anna Munke, Janina Sprenger, Guillaume Pompidor, Helena Taberman, Andrey Gruzinov, Jan Meyer, Johanna Hakanpää, Martin Gasthuber

**Affiliations:** a Deutsches Elektronen-Synchrotron DESY Hamburg Germany; b Center for Data and Computing in Natural Science CDCS Deutsches Elektronen-Synchrotron DESY Hamburg Germany; c Center for Free-Electron Laser Science CFEL Deutsches Elektronen-Synchrotron DESY Hamburg Germany; d Linac Coherent Light Source SLAC National Accelerator Laboratory Menlo Park USA; e The Hamburg Centre for Ultrafast Imaging, Hamburg, Germany; Brookhaven National Laboratory, USA

**Keywords:** macromolecular crystallography, serial crystallography, X-ray crystallography, real-time data processing

## Abstract

We report the use of streaming data interfaces to perform fully online data processing for serial crystallography experiments, without storing intermediate data on disk. The system produces Bragg reflection intensity measurements suitable for scaling and merging, with a latency of less than 1 s per frame. Our system uses the *CrystFEL* software in combination with the ASAP::O data framework. In a series of user experiments at PETRA III, frames from a 16 megapixel Dectris EIGER2 X detector were searched for peaks, indexed and integrated at the maximum full-frame readout speed of 133 frames per second. The computational resources required depend on various factors, most significantly the fraction of non-blank frames (‘hits’). The average single-thread processing time per frame was 242 ms for blank frames and 455 ms for hits, meaning that a single 96-core computing node was sufficient to keep up with the data, with ample headroom for unexpected throughput reductions. Further significant improvements are expected, for example by binning pixel intensities together to reduce the pixel count. We discuss the implications of real-time data processing on the ‘data deluge’ problem from recent and future photon-science experiments, in particular on calibration requirements, computing access patterns and the need for the preservation of raw data.

## Abstract 2

We report the use of streaming data interfaces to process data in real time from serial crystallography experiments, with a latency of less than 1 s per frame and without requiring intermediate data storage on disk.

## 1. Introduction

Serial crystallography experiments are well known for generating large amounts of data (Maia & Hajdu, 2016 ▸). These data sets consist of many thousands of images, each one being a single diffraction snapshot from an individual crystal. The usual processing pipeline for this type of data treats each frame on its own, and involves analysing the image to detect Bragg peaks followed by determining the orientation of the crystal based on the peak locations. This step is known as *indexing* the diffraction pattern. If it is successful, the next step is to calculate the expected locations of Bragg reflections based on the crystal orientation, and finally to measure the intensities of the Bragg reflections at their expected locations (including reflections which were too weak to be detected by the initial peak search). The intensity measurements are later combined into a single merged data set from which an electron-density map or other structural information can be derived.

Rapid feedback during the experiment is essential to enable the optimization of experimental parameters and to make the best use of limited beamtime. Online data processing has been available since the early years of serial crystallography (Foucar *et al.*, 2012 ▸; Foucar, 2016 ▸), and is now considered to be indispensable for a successful experiment. Online feedback systems analyse a sample of the data as it passes through the computer system. To date, they have been limited to simple diagnostics corresponding to the first stages of the crystallo­graphic processing pipeline, for example finding Bragg peaks and reporting the fraction of frames which contain plausibly useful diffraction patterns (the ‘hit rate’). Recently, GPU and FPGA hardware has been used to increase the speed of these systems, which allows a greater fraction of the data to be inspected and therefore raises the precision of the real-time information. However, online monitoring systems still concentrate on the peak-search and hit-finding stages (Leonarski *et al.*, 2023 ▸). Extending the online system to cover the complete processing pipeline, from detector readout images up to indexed Bragg intensity measurements, would open up many new possibilities. To do so requires increasing the speed of computationally intensive parts such as indexing, refinement and integration. Previous work in this area used supercomputing resources to attack the problem through massive parallelism (Blaschke *et al.*, 2021 ▸), but suitable supercomputing resources are not available close to all X-ray light-source facilities.

Processing the data in real time would allow electron-density maps to be made available during the experiment. This would be particularly beneficial for sensitive time-resolved pump–probe experiments, where the signal being studied is often a very small difference electron density. For a successful experiment, more feedback is needed than just hit rates: the clarity of the electron-density map needs to be inspected, and corrections possibly made to parameters such as the pump-laser alignment (spatial and temporal) or power. Without this feedback, problems which destroy the pump–probe signal altogether may not be detected until after the experiment. Worse still, the only avenues for ‘improvement’ of the signal will be changing the data-processing parameters, regardless of which aspects of the experiment truly needed improvement. Repeated reprocessing of data in search of a weak signal, when combined with publication bias, may raise questions about the reliability of the final results. For example, if a particular data set is repeatedly processed with different parameters, the electron-density map from each new processing run may contain different features at low significance, due simply to random noise. It could be very misleading to select (by stopping the reprocessing at that point) the result where the artefacts most resemble some expected pattern.

The next generation of free-electron laser sources are based on superconducting linear accelerators, which can produce more than a million pulses per second. Faster detector systems are also becoming available at synchrotron facilities, in particular at fourth-generation synchrotrons, such as the JUNGFRAU system, which can sustain a rate of 1000 frames per second including all of the required calibration steps (Leonarski *et al.*, 2023 ▸). The data rate is also increasing due to improvements in the efficient use of measurement time, for instance more reliable sample-delivery methods which allow data acquisition to continue without breaks (Oberthuer *et al.*, 2017 ▸) or crystal targeting systems which avoid the acquisition of blank frames (Oghbaey *et al.*, 2016 ▸). While the number of diffraction patterns for one structure may not increase, these improvements increase the total amount of data acquired, and almost invariably stored, by the facility as a whole. X-ray facililties are already beginning to impose severe restrictions on the amount of data allowed per experiment. In the context of these struggles, an even more dramatic and ambitious possibility enabled by full real-time data processing is that the intermediate data storage could be dispensed with altogether. This suggestion raises many questions about the underlying motivation for storing data, which will need to be addressed.

In this paper, we report a highly automated system for real-time data processing at the P11 beamline of the PETRA III synchrotron at DESY in Hamburg, Germany. The serial crystallography data-processing pipeline was made sufficiently fast to keep up with the data rate of a Dectris EIGER2 X 16 megapixel detector in real time, even with only a single computing node. Our data-processing system is based on the ASAP::O platform, described in Section 2, combined with the *CrystFEL* software for serial crystallography (White *et al.*, 2012 ▸). The key developments to improve the speed of *CrystFEL* are described in Sections 3 and 4. In Section 5, we describe the experiences from running this system over a series of user beamtimes between 2021 and 2023, including performance measurements which show that the system is likely to be scalable up to thousands of frames per second. Finally, we consider the possibilities, risks and implications of real-time data reduction, with a view to starting a discussion within the wider community.

## 2. The ASAP::O data system

ASAP::O (https://asapo.pages.desy.de/asapo) is a cutting-edge, high-performance distributed data-streaming platform that has been developed to meet the demands of both online and offline analysis for photon-science experiments at DESY. The ideas behind ASAP::O are quite similar to those of Apache Kafka (https://kafka.apache.org/) and similar streaming solutions, but ASAP::O has been developed and tuned for scientific use cases with their specific workflows and where the size of the messages is much larger: megabytes or gigabytes, compared with kilobytes in a traditional system.

The key capabilities of ASAP::O are as follows.

(i) High-performance and fault-tolerant delivery of data from an experimental data source (for example an X-ray detector) into a data-storage system.
(ii) Enabling different modes of data consumption, including random access, low-latency streaming and parallel access by multiple consumers.
(iii) Creation of computational pipelines by applying transformations to existing data streams.

To provide these capabilities, ASAP::O has three main components: a Producer API to send data into the system, a Consumer API to retrieve data from the system and a set of ASAP::O core services that run in the background on a single node or a cluster, depending on performance requirements. User programs do not need to be aware of the details of the core services, but rather use Consumer/Producer APIs in Python, C or C++.

Data within ASAP::O follow a structured hierarchy:

(i) Beamtime. The highest level, encompassing all data from a particular synchrotron experiment. Each beamtime is identified by a unique name which is ultimately generated by the proposal submission system.
(ii) Data source. Within each beamtime, various sources produce data, such as detectors or user applications. Each data source also has a name, unique within the beamtime, for identification.
(iii) Data stream. Each data source can generate multiple data streams, each with a unique name within that specific data source, which can be used to represent a ‘run’ of data acquisition.
(iv) Message. Data streams consist of flexible entities called messages, which contain metadata and binary data such as a detector image in a suitable data format such as HDF5. ASAP::O treats data as an opaque binary ‘blob’ and is not concerned with the format of the contents.

This scheme is quite flexible and allows for adaptations to the specific needs of experiments. In the work described in this article, we have only one data source – the area detector for X-ray diffraction; however, other data sources could exist in parallel. We use ASAP::O streams to represent user-initiated data-acquisition runs, each around 20 min, forming convenient units for record-taking. Each ASAP::O message within a stream contains the data for one image frame.

After defining a data scheme, a set of user programs has to be created to process data. Producer clients are responsible for creating data streams (*i.e.* for ingesting data into the system). Consumer clients are responsible for processing streams of data that were created by producers. A program that implements one step of a processing pipeline will read a data stream, process the data and send the results into another data stream, and so will simultaneously be both a consumer and a producer.

To apply *CrystFEL* to the data handled by ASAP::O, we implemented an ASAP::O consumer interface within the *CrystFELindexamajig* tool (White *et al.*, 2012 ▸). Instead of providing a list of files to be processed, we provide the ASAP::O endpoint address, data-source name, stream name and security token as command-line arguments. *Indexamajig* performs a peak search, indexes and integrates the frames, writing its output to disk as usual.

Our real-time data-processing system does not rely on storing data files, but we considered it important to enable a gradual migration from the current disk-based way of working. We therefore created a tool which stores the data in NeXus format (Könnecke *et al.*, 2015 ▸). This tool is written in Python, and creates a separate set of files for each ASAP::O stream. The frames are grouped together in batches of 1000 to produce a manageable number of reasonably sized files. A top-level ‘master’ file is also created, which uses the HDF5 virtual data-set functionality to link the batch data files together and add metadata such as the X-ray wavelength. This allows the entire data for one run to be accessed as if it were contained in one single file. Division into files also allows multiple slices of data to be written simultaneously, without an extra level of synchronization, which is not normally possible with HDF5,^1^ although parallelization was not found to be necessary in our case. Our chosen message format (see Section 5) meant that the ASAP::O data can be directly written to the HDF5 file without decompression and recompression, further increasing performance.

ASAP::O offers a level of fault tolerance. Data passing through the system are stored in a volatile memory cache, limited only by the available RAM of the ASAP::O servers. In the current deployment, in use as described later in this article, about 20 min of data could be stored. If, for example, the NeXus writer task were to crash, we would have 20 min to notice the problem and restart the writer, and perhaps pause data acquisition to allow it to catch up. A catch-up period might not be necessary in practice, since the file writing usually runs at a higher rate than the data acquisition. ASAP::O can additionally be configured to store the messages internally in a filesystem (which could consist of fast solid-state flash drives), which would give an even higher level of reliability, but the RAM cache combined with the NeXus writer program was found to be sufficiently reliable for this work.

Comparable systems for real-time data handling at other X-ray light-source facilities are often based on the message-queue system ZeroMQ. This is simple to get started with, but quickly becomes difficult as the pipeline grows more complex and important features are missed and need to be reimplemented. One such feature is that ASAP::O can automatically divide data between multiple consumers within a so-called ‘consumer group’ while allowing consumers to join and leave without losing data. This means that no extra code is needed in the analysis program to spread the computation between threads, processes or even separate computers. It is sufficient for them to simply request data from the same stream while declaring themselves all to be members of the same ASAP::O consumer group. This feature allows the computing resources to be dynamically scaled: if a program falls behind with its processing, additional instances of the program can be started on the fly. If too many instances are running, wasting computing resources, instances can easily be stopped and the work will be shared between the remaining instances. This setup can also be created using ZeroMQ; however, ASAP::O also allows multiple separate consumer groups to operate in parallel, with different access patterns, and without the groups and their access patterns being known in advance. For example, one consumer group could consist of multiple instances of an analysis process, while another consists of a single data-archiving process, and yet another consists of a live viewer which accesses only the most recent data. This type of pipeline structure closely matches our requirements, but is not directly supported by ZeroMQ.

**Figure 1:**
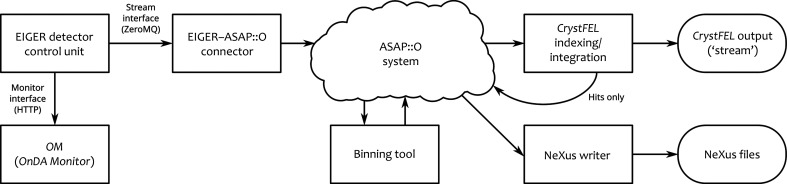
Overall flow of data through the software components of the system.

The overall flow of data through our system is shown in Fig. 1 ▸. To illustrate the possibilities, the diagram also includes two foreseen improvements: a binning tool and the option for *CrystFEL* to create a new ASAP::O stream containing only the ‘hit’ frames. These components are still under development, and their potential future importance is discussed in Sections 6 and 7.

## 3. Performance improvements within *CrystFEL*

Processing serial crystallography data sets has so far been thought of as a computationally demanding task. However, many speed improvements have been made in *CrystFEL* since version 0.10.0, which have combined to make real-time processing practical. Some of these improvements are changes to the code, and others are changes in the way that *CrystFEL* is used. We performed rounds of profiling (see Section 5) to find performance problems and fix or work around them. The most significant speed improvements are described in this section.

Firstly, the processing parameters for *CrystFEL* were selected to avoid excessive work per frame. *CrystFEL* was configured to use only one indexing algorithm, rather than a succession of alternative methods, and not to retry indexing if the initial attempt failed. The usual behaviour is to retry up to five times, each time deleting the weakest few peaks from the peak-search results. This improves the success rate, but obviously takes much more time. Many indexing algorithms are available within *CrystFEL*, and we performed a brief preliminary experiment to compare indexing success rates and average processing times, based on processing a sample of data from the previously deposited data set CXIDB-21 (Liu *et al.*, 2013 ▸; White, Barty *et al.*, 2016 ▸). We found that the *asdf* indexing algorithm gave the best overall trade-off between speed and success rate. Despite having been available for many years, the *asdf* algorithm has not been described previously in the literature, and therefore is described here in Section 4.

In addition to the run-time parameters for *CrystFEL*, it was important to ensure that the correct compile-time parameters had been chosen. For example, we realized that compiler optimizations had not been enabled for some data-compression components (the HDF5 ‘External filter plugins’ package), and enabling them approximately halved the time taken to read and decompress the image data from 122 to 59 ms for a 16 megapixel frame read from an HDF5 file on disk.^2^

We noticed a speed problem due to the method used for masking regions of the detector, such as the region shadowed by the beam stop. In *CrystFEL*, it is possible to define masked regions in several ways. In one of these ways, a rectangular region of pixels is defined with reference to the data axes of the image arrays, which would be appropriate for masking an area of noisy or otherwise defective pixels. Another option in *CrystFEL* is to define the region using coordinates in the laboratory frame, which would be appropriate for masking the pixels shadowed by a beam stop. However, this means that the range of pixels masked will change if the detector is moved. When masking pixels in this way, *CrystFEL* checks the laboratory-frame coordinates of all pixels on the detector to see if they fall within the masked area. For a 16 megapixel detector, this was found to take about a third of the total processing time, and the speed was improved by converting the expression of the masked regions to use pixel ranges instead of laboratory coordinates. A more efficient algorithm for selecting the masked pixels from laboratory-coordinate ranges is possible, using geometrical intersections, but has not yet been implemented.

We next turned our attention to the peak-search and indexing algorithms. The *peakfinder*8 algorithm (Barty *et al.*, 2014 ▸) works by calculating the statistics of pixel intensities in thin annular regions, to account for the much larger variation of background intensities in the radial direction compared with the circumferential direction. The assignment of pixels to annular regions usually does not vary between frames, so we modified the *CrystFEL* implementation of the *peakfinder*8 algorithm to pre-calculate the assignments once for all frames. We measured the average time saved by this as 376 ms per frame, under the conditions described above. This is only possible if the detector geometry is completely known in advance; the alternative, which is possible with *CrystFEL*, is to use per-frame metadata for values such as the beam centre or camera length. This grants additional flexibility, but means that the true detector geometry cannot be assumed to be constant between frames and is not completely known until each image has been loaded. We were therefore careful to ensure that the *CrystFEL* geometry file did not contain any such references.

To further increase the speed of the peak search, we reduced the number of pixels considered when calculating the mean and variance of the pixels within each annular region. The previous behaviour was to calculate the mean and variance of the pixel intensities from all pixels in the region. For a 16 megapixel detector, this is an excessively large number of pixels, and we found that adequately precise values could be obtained from only 100 randomly chosen pixels in each region. Measured again as described above, this reduced the total peak-search time from 178 to 75 ms per frame. We compared the results for a selection of images using the *CrystFEL* graphical user interface, and did not find any significant differences in the ability of the algorithm to find real peaks. However, since this change may slightly change the results of the peak search, we made the new behaviour optional and added a new command-line option (--peakfinder8-fast) to enable it.

Another inefficiency was found in the *CrystFEL* routine for calculating scattering vectors for reflections. In the old version, the matrix of unit-cell basis vectors was inverted on every call to the routine and the scattering vector calculated using trigonometric functions. In the new version, the result of the inversion is stored, and the vector is calculated by a matrix multiplication using the reciprocal Cartesian representation of the unit cell. This avoids both the repeated inversion and the computationally expensive use of trigonometric functions; however, it required some modification in the code because the program’s representation of a unit cell could no longer be considered as an immutable data structure (which is otherwise preferable for clean and memory-safe software design).

Finally, we discovered that the time taken per frame often increased by approximately 50% after processing a few tens of thousands of frames under the conditions described in Section 5. We do not yet have a complete explanation for this, and have already checked carefully to exclude a simple memory leak. We suspect that the slowdown is due to the behaviour of the operating-system kernel when allocating fresh memory many times across multiple processes. The previous behaviour of *CrystFEL* was to free all of the memory that it needed to process one frame immediately before starting work on the next frame, and reallocating approximately the same amount of memory. We modified *CrystFEL* such that each worker process used the same memory arrays for the image data and bad pixel masks for all the frames that it processed, which appeared to resolve this problem.

None of the improvements described in this section are specific to online data systems, because the adaptations made to allow *CrystFEL* to receive data via ASAP::O affected only the parts of the program which ingest data. Therefore, all of the efficiency gains will also increase the speed of traditional file-based processing.

## 4. The *asdf* indexing algorithm

Many indexing algorithms have been devised for the specific attributes of serial crystallography data (Ginn *et al.*, 2016 ▸; Gevorkov *et al.*, 2019 ▸, 2020 ▸; Beyerlein *et al.*, 2017 ▸; Brewster *et al.*, 2015 ▸; Li *et al.*, 2019 ▸). To date, the primary aim of these developments has been to obtain the highest success rate, and the time taken by the indexing algorithm has been a secondary concern. The applicable trade-offs are different for a real-time processing system: speed is of the highest importance, and a slightly lower success rate is acceptable if the algorithm completes several times faster. Many indexing algorithms are implemented within *CrystFEL*, and the current state-of-the-art algorithm, at least according to success rate, is *Xgandalf* (Gevorkov *et al.*, 2019 ▸). This algorithm typically completes in under 3 s, but this is too slow for real-time processing. The success rate of indexing depends on many factors, such as the unit-cell axis lengths: various cutoff values inside an algorithm might be tuned for larger unit cells, and not be optimal for smaller unit cells. The speed of indexing can also depend on these factors, for similar reasons: one algorithm may ‘give up’ on a certain pattern earlier than another, based on a cutoff value.

For this work, we found that the best compromise between indexing success rate and speed was the *asdf* algorithm, which completes in under half a second but still with a high success rate. The *asdf* algorithm was added to *CrystFEL* many years ago in version 0.6.1 (released in August 2015), but has not yet been described in the scholarly literature. It is essentially a reimplementation of the *DirAx* algorithm (Duisenberg, 1992 ▸), but using a fast Fourier transform for the one-dimensional periodicity search at the core of the algorithm. It additionally includes a unit-cell volume constraint, which filters out bad indexing solutions early, based on the known unit-cell parameters. Since *asdf* is implemented directly within *CrystFEL*, there is no need to create temporary files, run a separate program or parse output, as is the case when using external programs such as *MOSFLM* (Powell, 1999 ▸), *DirAx* (Duisenberg, 1992 ▸) or *XDS* (Kabsch, 1988 ▸) as the indexing engine.

Briefly, the *asdf* algorithm operates as follows.

(1) Calculate the three-dimensional reciprocal-space coordinates corresponding to all spots found by the peak search, based on the Ewald sphere construction using the nominal radiation wavelength and ignoring any wavelength spread.
(2) Assemble the reciprocal-space points into groups of three (triplets). If the number of triplets is very large, randomly select 20 000 triplets based on the first 2000 reciprocal-space points. Otherwise, generate all of the possible triplets.
(3) For each triplet, perform the following steps.
  (*a*) Calculate the normal to the plane containing the three points. Project all reflection positions onto a line through the origin in this direction to produce a set of distances from the origin.
  (*b*) Create a one-dimensional array of 1024 real-valued elements, containing zeroes. The first element of the array will correspond to the most negative distance determined in the previous step, whereas the last element will correspond to the most positive distance. The elements in between will correspond linearly to the distances in between the two. For each of the distances, add 1 to the corresponding element of the array.
  (*c*) Perform a Fourier transform of the array. This produces a new one-dimensional array with complex-valued elements, each of which corresponds to a separation length between the reflection positions.
  (*d*) Find the element of the transformed array with the largest magnitude. Calculate the length that the index of this element corresponds to. Note that the reciprocal of this length, in the direction of the normal vector for this triplet, is a candidate for one of the direct-space lattice vectors.
  (*e*) Determine which of the distances from step (*a*) are close to integer multiples of this length. Using these distances, perform a least-squares fit to estimate the repeat length more accurately.
  (*f*) After the least-squares fit, if more than six points are close to integer multiples of the estimated distance, accept the candidate lattice vector. Otherwise, disregard it from further processing.
(4) Determine the three shortest linearly independent vectors with sufficient fits, and construct a unit cell from them. If prior information is available about the lattice parameters, require that the volume of the cell produced here matches the volume of the reference unit cell.
(5) Return the unit cell for the prediction, integration and output stages of the pipeline (White, Mariani *et al.*, 2016 ▸).

During the course of this work, further speed improvements have been made to *asdf*. We added the limit on the number of triplets in step (2) after noticing the excessive amount of calculation from our profiling data described in Section 5. We later reduced the limit on the number of triplets from 20 000 to 10 000 and the number of reflections considered from 2000 to 120. We tested this change on a sample of 1000 EIGER 16M frames in a NeXus file on disk, using an eight-core Intel Xeon W-2225 processor at 4.1 GHz clock frequency and 16 Gb of memory, with *indexamajig* running as a single thread. The data files came from one of the experimental runs described in Section 5. Restricting the triplet search parameters increased the average speed of the *asdf* algorithm from 318 ± 219 ms to 106 ± 49 ms. In this test, there was no decrease in the indexing success rate (144 indexed frames out of 576 ‘hits’ within the 1000 test frames), but since the reduction may theoretically change the results, we made this change optional by adding a new option to *indexamajig* (--asdf-fast) to preserve the compatibility with previous results.

## 5. Beamtime experiences and performance evaluation

We have extensively tested the real-time data-processing system at the macromolecular crystallography beamline P11 at PETRA III, DESY, Hamburg, in multiple experiments since September 2021. For these tests, we used the standard detector at the beamline, which is a Dectris EIGER 2X detector with 16 megapixels. The experiments were carried out using the CFEL tape-drive system for crystal delivery (Zielinski *et al.*, 2022 ▸).

To connect the EIGER detector at P11 with ASAP::O, we wrote a Python program which connects to the EIGER detector’s ZeroMQ streaming interface and passes the data into ASAP::O. We initially used HDF5 for the data format.^3^ However, the flexible data model of HDF5 is superfluous for our purposes, and it is sufficient to transfer a single data array, provided that some basic information is included about the array dimensions and data type. The EIGER streaming interface already provides data compressed with LZ4+Bitshuffle, which we used directly as the payload of the ASAP::O messages. We wrote a new serialization library, called Seedee, to abstract the data format and compression algorithm details within *CrystFEL* and other tools using the system. The EIGER–ASAP::O connector program runs continuously, starting a new ASAP::O stream whenever the detector reports a new run number. The run number itself is generated by the beamline control software, and is sent to the EIGER detector control unit using its HTTP ‘Simplon’ interface for inclusion in the message headers.

To evaluate the performance of the system, we acquired data from lysozyme crystals using the JINXED crystallization method (Henkel *et al.*, 2023 ▸). Since these crystals are a well known standard, they allowed us to control the hit rate. We processed the unbinned full-frame readout from the detector, running at its maximum possible speed of 133 frames per second. The experimental conditions were monitored using *OnDA Monitor* (Mariani *et al.*, 2016 ▸) connected to the HTTP monitoring interface of the EIGER detector, completely separate to the ZeroMQ interface used for the real-time processing. We held the hit rate close to 100%, meaning that the number of blank frames was low compared with a ‘real’ sample. Since blank frames can be skipped over soon after the peak search, this provided a more severe test of the performance of the indexing and integration.

As usual for serial crystallography experiments, measurements were made in ‘runs’ containing between 10 000 and 400 000 frames, or between 1.25 and 50 min at 133 frames per second. For the first experiments, we configured *CrystFEL* to automatically move between acquisition runs, producing one large output file. However, we quickly found it better to preserve the separation into runs for the online system, which makes it easier to spot differences in behaviour of the sample or the processing system. To this end, a new *CrystFEL* process was started for each run, writing to a new output file and producing separate log files. A web-based database system stored the processing parameters, monitored the output and presented the results, as well as providing an interface for merging the data and calculating electron-density maps.^4^

We modified the *CrystFELindexamajig* tool to measure the time taken by various steps of processing. This was performed by adding instrumentation code which measured the time elapsed between the start and end of various segments of code. The ‘wall clock’ time was used, rather than the cumulative amount of run time allocated to the program by the operating system, in order to explicitly include periods such as when the operating system suspended execution of the program while waiting for network data. One record was written for each iteration of the main processing loop inside *indexamajig* — requesting an image frame, processing it and writing the results — regardless of whether the full processing arc was completed or broken off early. Each record consisted of a hierarchical tree structure, where each node was linked to lower-level nodes which account for the time taken by instrumented code segments encountered while the ‘parent’ segment was still active. For example, subtasks for loading the image data include allocating memory, decompressing the data and setting up certain metadata structures. The total time taken to process the image (which was labelled as root), always forms the ‘trunk’ of the tree. Initially, the instrumentation was added at the level of the main processing tasks, such as loading data, peak search or indexing. Lower-level segments were instrumented once we ascertained which parts consumed the most time. This performance profiling system has already been used independently by another group and has been described by them (Gasparotto *et al.*, 2024 ▸).

**Figure 2:**
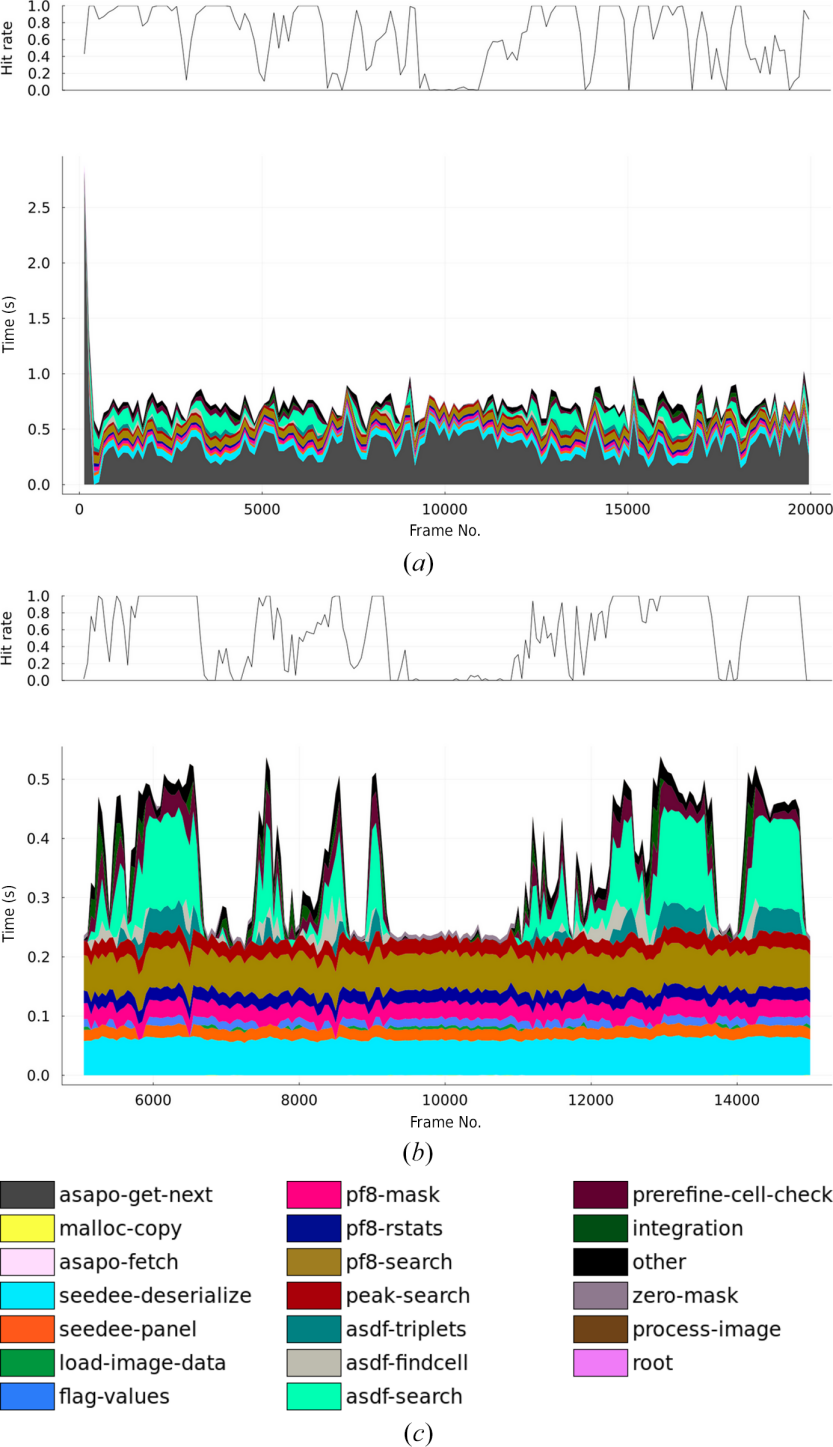
Performance profiling results during a run with a high hit rate. (*a*) The first 20 000 profiling records, averaged in blocks of 133 records. A longer wait period is visible for the very first frames, after which the average processing time (including wait time) closely matches the available time per frame (722 ms). (*b*) Expanded view of records 5000–15 000, during which the hit rate dropped to zero for a short period. The wait time has been removed to more clearly show the true processing time per frame. Records were averaged in batches of 50. (*c*) Colour key.

To plot the profiling results, we created a visualization package in the Julia programming language. We first averaged the times in groups (of 50 or 133 records, as noted in the figure captions), taken in order of the completion time for each iteration of the processing loop. For each batch of records, we took the ten largest times, and grouped the remaining times into a single other group. The groupings were then plotted as a stacked area plot [Figs. 2 ▸(*a*) and 2 ▸(*b*)]. The colour key is shown in Fig. 2 ▸(*c*), which includes all timing records not grouped into the other category in either graph. To fully understand the meanings of the timing definitions requires inspection of the *CrystFEL* code, because they depend on the implementation details of the algorithms. However, they can be briefly explained as follows.

asapo-get-next. Requesting the next frame from the ASAP::O system.

malloc-copy. Allocating a memory block for intermediate storage of the ASAP::O data.

asapo-fetch. Catch-all group for any remaining time taken during retrieval of the data from ASAP::O.

seedee-deserialize. Decompressing the image data.

seedee-panel. Converting the image data to a standard format (IEEE 754 single-precision floating point) while copying the data into image-panel data locations.

load-image-data. Catch-all group for any remaining time taken during loading of image data into *CrystFEL* data structures.

flag-values. Marking bad pixels according to their values in the image data. In this case, any pixel with a value of 65 535 is considered masked.

pf8-mask. Preparing internal data structures for the *peakfinder*8 algorithm.

pf8-rstats. Calculating the mean and variance of the pixel values in annular regions, part of the *peakfinder*8 algorithm.

pf8-search. Comparing the pixel values with the mean and variance, and calculating the peak locations, within the *peakfinder*8 algorithm.

peak-search. Catch-all group for any remaining time spent in the peak-search algorithm.

asdf-triplets. Generating the reciprocal-space triplets; part of the *asdf* indexing algorithm.

asdf-findcell. Attempting to assemble a unit cell out of combinations of the potential basis vectors within the *asdf* algorithm.

asdf-search. Catch-all group for any remaining time spent in the search phase of the *asdf* algorithm. This includes the time taken for the 1D Fourier transforms of the projected spot positions.

prerefine-cell-check. Comparing the unit cell produced by the indexing algorithm against the reference cell parameters.

integration. Calculating (‘predicting’) the reflection positions based on the indexing solution, and measurement of the intensities from the image.

zero-mask. Initializing the bad pixel mask to zero (see the note at the end of Section 3).

process-image. Catch-all group for any remaining time spent working on an image (not the time spent between processing images).

root. Catch-all group for any remaining time not covered by the groups above (and also not falling into the other category; see above).

Fig. 2 ▸(*a*) shows timing data from a run with an overall average hit rate of 54%. The processing was run with 96 worker processes on a single 96-core compute node (192 virtual processors when including simultaneous multi-threading). Note that not all categories are clearly visible in the plots: malloc-copy is visible as a very thin strip at the bottom of Fig. 2 ▸(*b*), whereas asapo-fetch, process-image and root are not visible at all. Their descriptions are nevertheless given for completeness, and the near-invisibility of the smallest times gives confidence that all relevant time has been visualized. The near-invisibility of the root category also supports this confidence.

The most time-consuming step is apparently asapo-get-next, in which *CrystFEL* requests the ASAP::O system to provide the next frame. However, this actually indicates that too many indexing workers were running for the situation, therefore the workers spent most of their time waiting for data. With 96 worker processes, on average 722 ms are available for processing each image (96 × 1/133). If the processing time for one frame is less than this, the program will have to wait for the next frame to become available. The average processing time across the whole run, including this waiting time, was 731 ms, in agreement with the expected value.

At the very start of the run, much longer waiting times are seen. These reflect the timeout-based behaviour of ASAP::O: if no data is available, the API call to get the next frame will return after a user-definable timeout, which was set to 3 s in this work. After the initial period, the next frames are processed with very little waiting time, while the system catches up with the cached data before the steady state establishes itself. The behaviour shown in the graph persisted for the entire duration of even the longest runs (400 000 frames, lasting 50 min), which corresponds to more data than can fit in the ASAP::O memory cache. This demonstrates that the processing system is stable under steady-state situations, not just under ‘burst’ conditions.

Excluding the asapo-get-next time from the graph reveals the true time taken by the processing, and how it strongly depends on the hit rate [Fig. 2 ▸(*b*)]. The average processing times for hits and non-hits in the entire run were 455 and 242 ms, respectively, and the overall average processing time was 378 ms. The most time-consuming step overall is seen to be the peak search, which is expected because of the large number of pixels and because the peak search must look at every pixel of every frame. For hit frames, the processing time is approximately doubled because of the long search for lattice vectors.

It is somewhat remarkable that the time for comparing unit-cell parameters (prerefine-cell-check) appears prominently enough to be seen on the graphs. This task appears trivial, but is complicated by the fact that the unit cell produced by the indexing algorithm can be an alternative representation of the correct unit cell. For example, a primitive representation might be produced for a centred unit cell. In general, there are an infinite number of possible representations for any lattice. Lattice representation and comparison is, even today, an active area of research (Andrews & Bernstein, 2023 ▸). *CrystFEL* is currently using an algorithm based on comparison of Niggli-reduced cells in the *G*^6^ space (Andrews & Bernstein, 1988 ▸), but recently described alternative algorithms, specific to the requirements of serial crystallography, may be faster (Andrews *et al.*, 2023 ▸).

Our experiments with the real-time system revealed the importance of understanding the interaction between elements of the high-performance computing environment in which it runs. In our first experiments, before implementing many of the optimizations described in Section 3, we had to split the *CrystFEL* processing across multiple compute nodes. After the speed improvements, a single node was found to be sufficient, even when sharing the computer with other tasks such as the *OnDA Monitor*. However, this meant that all of the data needed to flow over one network link to one computer, and the bandwidth of the link was not sufficient. It can, therefore, be better to spread the computing over multiple smaller computers, rather than to use a single very powerful one. This bottleneck led us in turn to detect a software library misconfiguration which caused data to flow over 10 gigabit per second ethernet links within the data centre, instead of 100 gigabit per second Infiniband links as intended. With the compressed size of each frame from the EIGER detector being about 7 megabytes, the total data rate is approximately 8.5 gigabits per second, and the 10 gigabit connection is not quite sufficient when including the co-existing *OnDA Monitor* as well as network overheads. This misconfiguration was fixed for later experiments.

**Figure 3:**
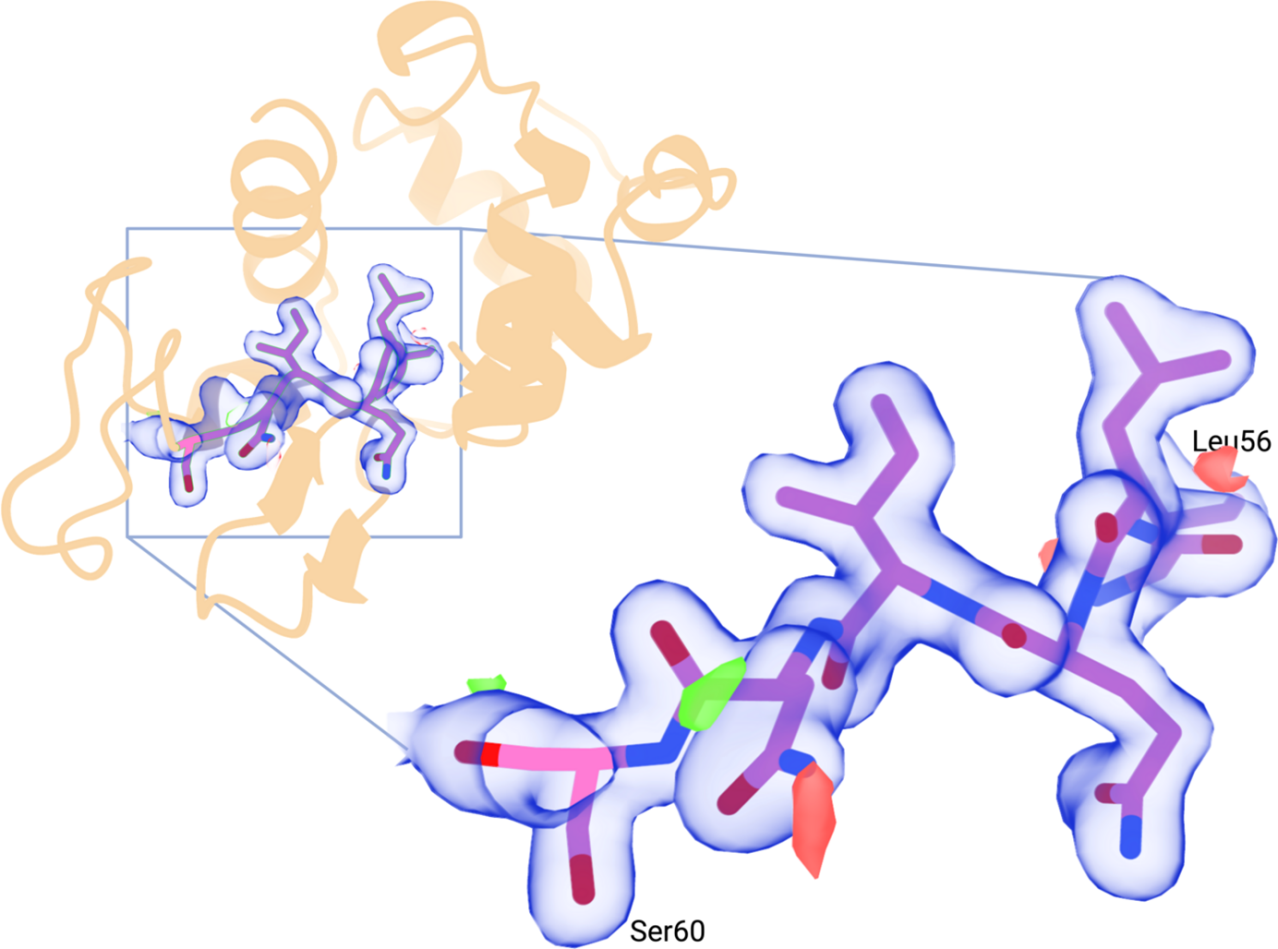
Overall structure (cartoon plot, orange) of lysozyme obtained after automated refinement and model building against data produced by the real-time processing system. Residues ranging from Leu56 to Ser60 are displayed as sticks and overlaid with a 2*F*_o_ − *F*_c_ map (blue, sigma of 1.0 and cutoff radius of 1.6 Å) and *F*_o_ − *F*_c_ maps (green, sigma of +3.0; red, sigma of −3.0; cutoff radius around the residues of 2 Å). This figure was created with BioRender.com.

To complete the validation of the pipeline, we report a protein structure solved using data from the real-time pipeline. We took a single run of 200 000 diffraction patterns from JINXED lysozyme crystals as described above, from which 14 322 lattices could be indexed and integrated by the pipeline. The *indexamajig* output from this run was merged using *partialator*. Partiality modelling was not used (the ‘unity’ model was selected in *partialator*). This resulted in a data set with useful resolution out to 1.8 Å, judged by the point where CC* fell below 0.5, with the entire resolution range to the corner of the detector divided into 20 shells. The correct space group was assigned and *R*_free_ flags were generated using the program *phenix.reflection_file_editor*. A previously deposited JINXED lysozyme model processed using the traditional offline analysis pipeline (PDB entry 8b3l) was used as a starting model for refinement after the removal of alternate conformers, waters and all nonprotein residues apart from one Na^+^ ion and two Cl^−^ ions. The unit-cell parameters from this structure, which were used as reference parameters for the indexing pipeline, were also used for refinement. Refinement was carried out using *phenix.refine* (Afonine *et al.*, 2012 ▸) and the results of each round of refinement were inspected using *Coot* (Emsley *et al.*, 2010 ▸), but we omitted all manual rebuilding steps. This was followed by a final automated refinement and rebuilding step using *PDB-REDO* (Joosten *et al.*, 2014 ▸), resulting in a model refined against data out to 1.8 Å resolution (*R* and *R*_free_ of 0.19 and 0.22, respectively; Fig. 3 ▸). The isotropic *B* factors are slightly higher than would usually be expected (34.62 Å^2^ compared with 25.04 Å^2^ for the previous JINXED model), which could be attributed to shortcomings in geometry optimization of the real-time pipeline and needs to be investigated further. Otherwise, no pathologies could be detected in the model. Data-processing and model statistics are shown in Table 1 ▸, and the resulting model was deposited in the PDB with accession code 8rpm.

## 6. Discussion

We begin this section by acknowledging the importance of preserving raw data. Important reasons for data preservation include the principles of basic scientific integrity and reproducibility, as well as the hope for future improved data-processing methods. In the past, the cost of data archiving has been negligible compared with the cost of repeating the experiment, taking into account the cost of protein expression, purification and crystallization, the costs of researcher travel to the X-ray facility, and the cost of operating the facility itself. However, the costs of large-scale data storage are huge, and now can be much greater than the other costs of the experiment. At current prices, the marginal cost of storing four petabytes of data for five years, on reliable enterprise-grade storage systems, is around 200 000 Euros. The European XFEL beamlines easily produce around a petabyte of data per day during operation. Some of the other experimental costs are decreasing: for example, travel is less necessary in view of improved systems for remote facility access and ‘mail-in’ samples. The data-storage costs are increasing, in contrast, and will make up a larger fraction of the overall costs.

Previously, storage of data on disk was an integral part of the processing pipeline: data files on disk formed the ‘connection’ between the data-acquisition and data-processing stages of an experiment. Our real-time system instead makes this connection using network links and in-memory systems. Data can be persisted on disk, but this function is optional and occurs at the *end* of the pipeline, not in the middle (see Fig. 1 ▸). We are therefore free to choose the manner of data storage appropriate to the scientific goals. Several scenarios can be envisaged, depending on the requirements of the experiment and the need for consideration for future improvements to the data processing. Possible considerations include reproducibility, requiring us to store only the frames which were successfully indexed and integrated (*i.e.* which contributed directly to the final merged data), but in raw format with no lossy compression or binning. Another consideration is ‘improvability’, which would require us to additionally store frames that could not be successfully processed, in the hope that they might become usable with future developments in analysis methods. Yet another consideration is integrity, for which we could store a much smaller sample of frames as proof that the results were not fabricated. In the event that a bug was found in the analysis software at a later date, a small data sample could allow us to check whether the experiment was affected and validate the conclusions.

Abandoning storage of all raw data will reduce the costs, but has obvious risks. Accurate calibration information is required for the detector geometry and its intensity response, and many data-processing problems in serial crystallography are attributable to inaccurate calibration. In this work, we used an EIGER detector, which has excellent properties in this regard: it requires no regular recalibration of gains, has a stable set of bad pixels and consists (from the software point of view) of a single large panel. Segmented detectors with adaptive gain switching, such as the AGIPD (Allahgoli *et al.*, 2015 ▸), ePix (van Driel *et al.*, 2020 ▸), CSPAD (Carini *et al.*, 2013 ▸) or LPD (Veale *et al.*, 2017 ▸), present much larger calibration challenges, which will need to be reliably and preferably automatically solved. Other possible risks are that the crystal lattice parameters do not match the reference parameters (which would prevent any diffraction patterns from being indexed), or that the peak-search parameters were set too conservatively (which, in an extreme case, could lead to hit frames being wrongly classified as blanks). Real-time feedback tools, using data from the real-time processing results, could be added in the future to mitigate these risks. Changes might be needed to the workflow of an experiment, such as taking time to carry out a short preliminary experiment to determine the lattice parameters if they are initially unknown.

At one extreme, a problem with the data processing could mean that the entire experiment would need to be repeated, including purifying and crystallizing the protein sample. The core question is whether the risk of this situation is acceptable, balanced against the cost of storing large amounts of data as a type of ‘insurance’. The acceptable level of risk will depend on the type of experiment. For example, an experiment probing very valuable crystals of a large unit-cell membrane protein complex will value the raw data much more highly than a pharmaceutical ligand-screening experiment probing thousands of very similar samples.

We would like to draw attention to the distinction between real-time *processing*, the main focus of this paper, and real-time *monitoring*. For the best user experience, the real-time processing system described here can be complemented by a monitoring system based on *OnDA Monitor* (Mariani *et al.*, 2016 ▸) in which the instantaneous hit rates and indexing rates are graphed continuously, independent of any partitions into runs. The *monitoring* system indexes patterns with no prior information and reports the observed lattice parameters, which allows any contaminants or alternative crystal forms to be quickly spotted. The real-time *processing* system, in contrast, indexes the patterns using prior information about the crystal lattice parameters, in order to ensure that the patterns are all indexed consistently and can be merged. Our real-time monitoring system can operate independently, taking data from the monitoring interface of the EIGER detector (which provides a low-speed stream with a small sample of the data), or via ASAP::O. We found it helpful to make this distinction during our experiments, rather than to combine both aspects into one system. The monitoring system will be described in a future publication.

Real-time data processing changes the usage pattern of high-performance computing resources. When data processing is considered to be a separate step, a researcher might submit a large array of jobs at the end of one day, and expect to see the results the next morning. The researcher need not be concerned with exactly when the computing cluster runs the analysis jobs, and the cluster’s load-management software is free to schedule the jobs in the most convenient way. Serial crystallography data-processing jobs can additionally be broken down into smaller independent jobs, say of around 1000 frames each, which makes the scheduling even easier: many small jobs can fill the available time amongst other jobs which require larger numbers of CPUs to be available simultaneously. With real-time processing during the experiment, sufficient resources must be available at the time of data acquisition. In principle, this appears to mean that dedicated nodes must be permanently allocated to each beamline. However, other modes of operation might be considered, such as sharing resources between beamlines and cooperatively scheduling the start times and lengths of acquisition runs to avoid contention.

## 7. Conclusion and outlook

We have reported a system for real-time processing of serial crystallography data. The system has been extensively tested at PETRA III, and has already become an indispensable part of the setup for serial crystallography at P11.

In future work, we plan to interpose a binning worker to reduce the 4148 × 4362 pixel size of the EIGER detector to 2074 × 2181, 1382 × 1454 or 1037 × 1090 pixels, by combining pixel readout values in squares with side lengths of two, three or four pixels, respectively. Here, we have tested with the full 16 megapixel detector resolution, but this is excessive for all but the largest unit-cell sizes. The binning worker has been implemented, but not yet thoroughly tested, and is shown in Fig. 1 ▸. It operates by reading the image data stream from ASAP::O and writing a new stream with the binned data. The performance profiling results show that most of the processor time is spent on per-pixel operations, so reducing the number of pixels is likely to produce a proportional increase in the speed.

We intend to test the system at the European XFEL, using either the AGIPD or LPD detectors. Compared with the reference experiment described in this paper, these detectors have 1/16 the number of pixels (one million compared with 16 million), a higher frame rate (3520 frames compared with 133 frames per second), more complicated geometry (16 and 64 panels, respectively, compared with one panel) and additional complications due to adaptive gain switching. We expect that real-time processing will be computationally feasible under these conditions, but the extension to segmented detectors will increase the need for rapid refinement of detector geometry. We therefore plan to additionally implement a system for continuous geometry refinement, which can be combined with the real-time processing system such that a refined geometry description is always available, with little to no user effort required.

We are also currently implementing and testing the option to store only the hits or the indexed frames. One option for this, shown in Fig. 1 ▸, is to create a separate ASAP::O stream from within *CrystFEL* and configure the NeXus writer tool to take its input from that, instead of from the stream written by the EIGER connector. This will be an important step towards realizing the full potential of real-time data processing.

Finally, we invite and look forward to discussion within the wider crystallography community about the most appropriate way to reduce the large data-storage costs, while simultaneously satisfying our standards for reproducibility.

## 8. Software and data availability

*CrystFEL* is free and open-source software available from https://www.desy.de/~twhite/crystfel/. The ASAP::O interface for *CrystFEL* has been included since version 0.10.2, and the *asdf* indexing algorithm was first included in version 0.6.1. These, as well as other relevant features, are being continuously developed, and the very latest versions are available from the version-control repository accessible via the website. We have collected recommendations for streaming and high-speed data processing within the *CrystFEL* source-code repository itself, as https://gitlab.desy.de/thomas.white/crystfel/-/blob/master/doc/articles/online.rst and https://gitlab.desy.de/thomas.white/crystfel/-/blob/master/doc/articles/speed.rst, respectively. The programs for analysing and plotting the *CrystFEL* performance profiling data, written in Julia, are available from https://gitlab.desy.de/thomas.white/profileanalysis.jl.

The ASAP::O framework is currently only deployed at DESY, but information is available from https://asapo.pages.desy.de/asapo/ and source code from https://gitlab.desy.de/asapo/asapo. The Seedee serialization library can be downloaded at https://gitlab.desy.de/fs-sc/seedee, and is also available via PyPI (https://pypi.org/project/seedee/). The EIGER–ASAP::O Connector and ASAP::O NeXus writer tools will be made publicly available soon. The complete system is available for use at P11, supported by the Scientific Computing group at DESY Photon Science.

The lysozyme structure has been deposited in the Protein Data Bank with accession code 8rpm.

## Supplementary Material

PDB reference: lysozyme structure from data processed in real time, 8rpm

## Figures and Tables

**Table 1:** Data and structure-solution statistics for the lysozyme structure Values in parentheses are for the outer shell.

PDB code	8rpm
Data-collection temperature (K)	295
No. of collected frames	200000
No. of hits	60317 [30.2% of frames]
Indexable frames	14322 [23.7% of hits]
Indexed lattices	14322
Space group	*P*4_3_2_1_2
*a*, *b*, *c* (Å)	79.200, 79.200, 38.000
α, β, γ (°)	90, 90, 90
Resolution (Å)	56.00–1.80 (1.864–1.800)
Unique reflections	11716 (1118)
〈*I*/σ(*I*)〉	5.292 (0.94)
Completeness (%)	100 (100)
Multiplicity	373.9 (248.5)
*R*_split_	0.117 (1.115)
CC_1/2_	0.98 (0.446)
Wilson *B* factor (Å^2^)	37.95
Resolution range used in refinement (Å)	56.00–1.80 (1.847–1.800)
Reflections used in refinement	10264 (586)
Reflections used for *R*_free_	637 (38)
*R*_work_/*R*_free_	0.185/0.222 (0.381/0.441)
R.m.s.d., bond lengths (Å)	0.005
R.m.s.d., angles (°)	1.433
Ramachandran favoured (%)	98.4
Ramachandran allowed (%)	1.6
Ramachandran outliers (%)	0
Average *B* factor (Å^2^)	34.619

## Footnotes

1 The necessary synchronization can be implemented using MPI (Message Passing Interface), but this would require a very different design for the program.

2 This speed measurement was performed in a single thread on a desktop computer equipped with an eight-core Intel Xeon W-2225 processor at 4.1 GHz clock frequency and 16 Gb of memory.

3 The HDF5 format can be used with data in memory, without creating files on disk.

4 The web-based database system, called ‘Amarcord’, has much larger scope and will be described in detail in a future publication.
